# RETRACTED: Kosumi et al. Systemic Retinoids for Generalized Verrucosis Due to Congenital Immunodeficiency: Case Reports and Review of the Literature. *Genes* 2023, *14*, 769

**DOI:** 10.3390/genes15080998

**Published:** 2024-07-30

**Authors:** Hideyuki Kosumi, Ken Natsuga, Teruki Yanagi, Hideyuki Ujiie

**Affiliations:** Department of Dermatology, Faculty of Medicine and Graduate School of Medicine, Hokkaido University, Sapporo 060-8638, Japan; yanagi@med.hokudai.ac.jp (T.Y.); h-ujiie@med.hokudai.ac.jp (H.U.)

The journal *Genes* retracts the article, “Systemic Retinoids for Generalized Verrucosis Due to Congenital Immunodeficiency: Case Reports and Review of the Literature” [1], cited above. 

Following publication, the authors raised the attention of the Editorial Office to concerns relating to the accuracy of the findings presented in this case report [1].

As indicated by the authors, the conclusions of this case report [1] are significantly dependent on the findings presented in an article [2] previously published by the same authorship group. As this earlier article [2] has now been retracted, the Editorial Board and authors have agreed that the findings of this case report [1] can no longer be sustained. Therefore, the Editorial Board has decided to retract this publication [1] as per MDPI’s retraction policy (https://www.mdpi.com/ethics#_bookmark30, accessed on 24 June 2024).

This retraction was approved by the Editor-in-Chief of the journal *Genes.*

The authors have agreed to this retraction.
